# 

**Published:** 1917-03

**Authors:** 


### CONTENTS.

ORIGINAL COMMUNICATIONS....................469

SELECTIONS AND ABSTRACTS....................479

BOOK REVIEW....................496

